# An unusual case of fatty posterior mediastinal ganglioneuroma

**DOI:** 10.1259/bjrcr.20150482

**Published:** 2016-11-26

**Authors:** Abdelrahman M. Abdelazim, Smita A Patel, Shawn Haji-Momenian, MI Almira-Suarez, M Reza Taheri

**Affiliations:** ^1^Department of Radiology, George Washington University, Washington, DC, USA; ^2^School of Medicine and Health Sciences, George Washington University, Washington, DC, USA; ^3^Department of Pathology, George Washington University Hospital, Washington, DC, USA

## Abstract

Ganglioneuromas, which arise from neural crest cells, are typically seen in adolescent and young adults. We describe an unusual case of posterior mediastinal ganglioneuroma with a large fatty component in a middle-aged male. This imaging feature has only been reported in five published manuscripts in the English literature.

## Case presentation

A 48-year-old male presented with a paraspinal mass seen on preoperative chest X-ray obtained for knee arthroscopy. He reported relatively constant sharp left axillary pain radiating to the anterior chest wall for about 8 months.

## Investigation

**Figure 1.**
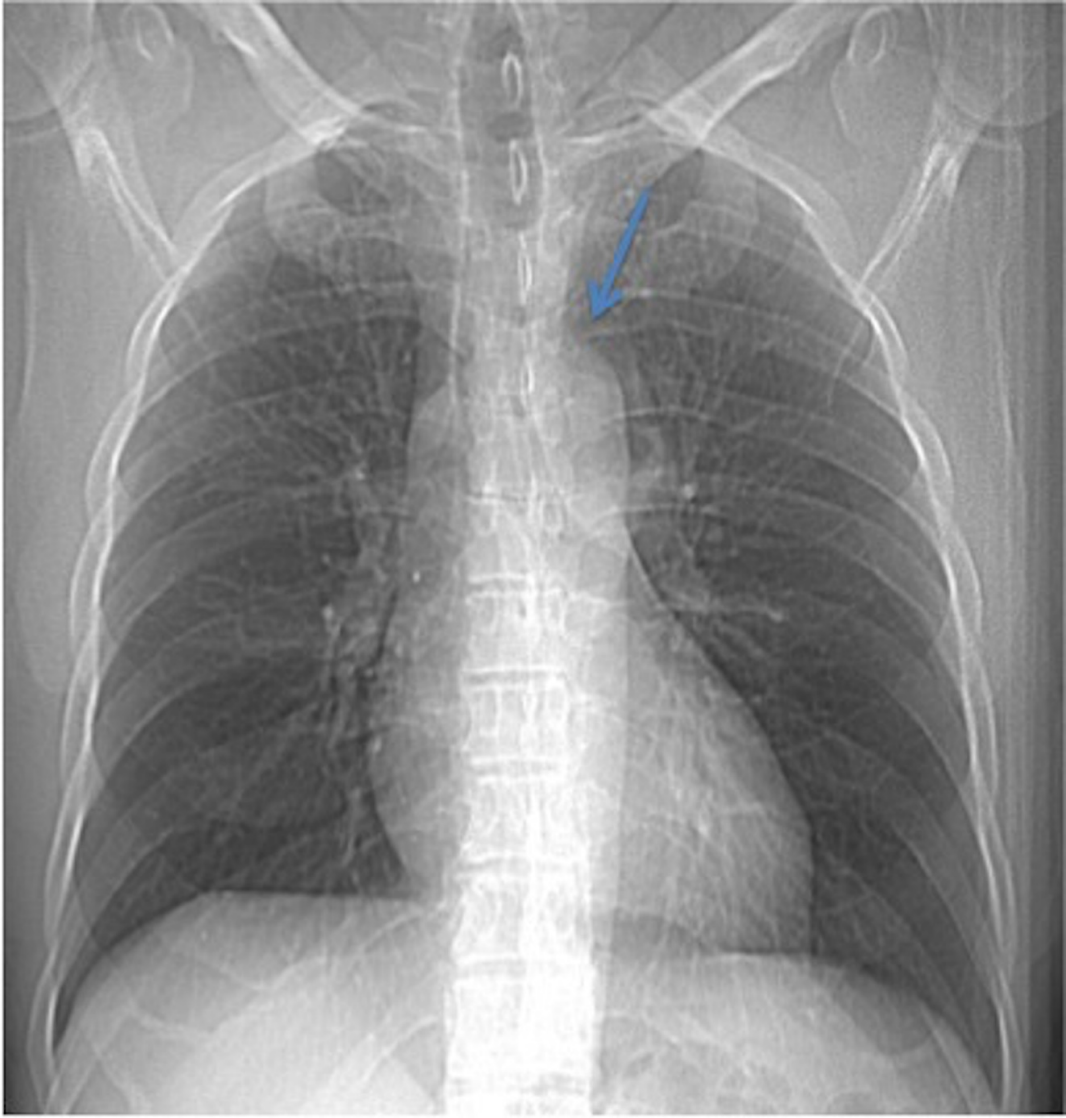
A scout film of the CT scan of the chest shows a paraspinal soft tissue density that obscures the left supralateral margin of the aortic arch (arrow).

**Figure 2.**
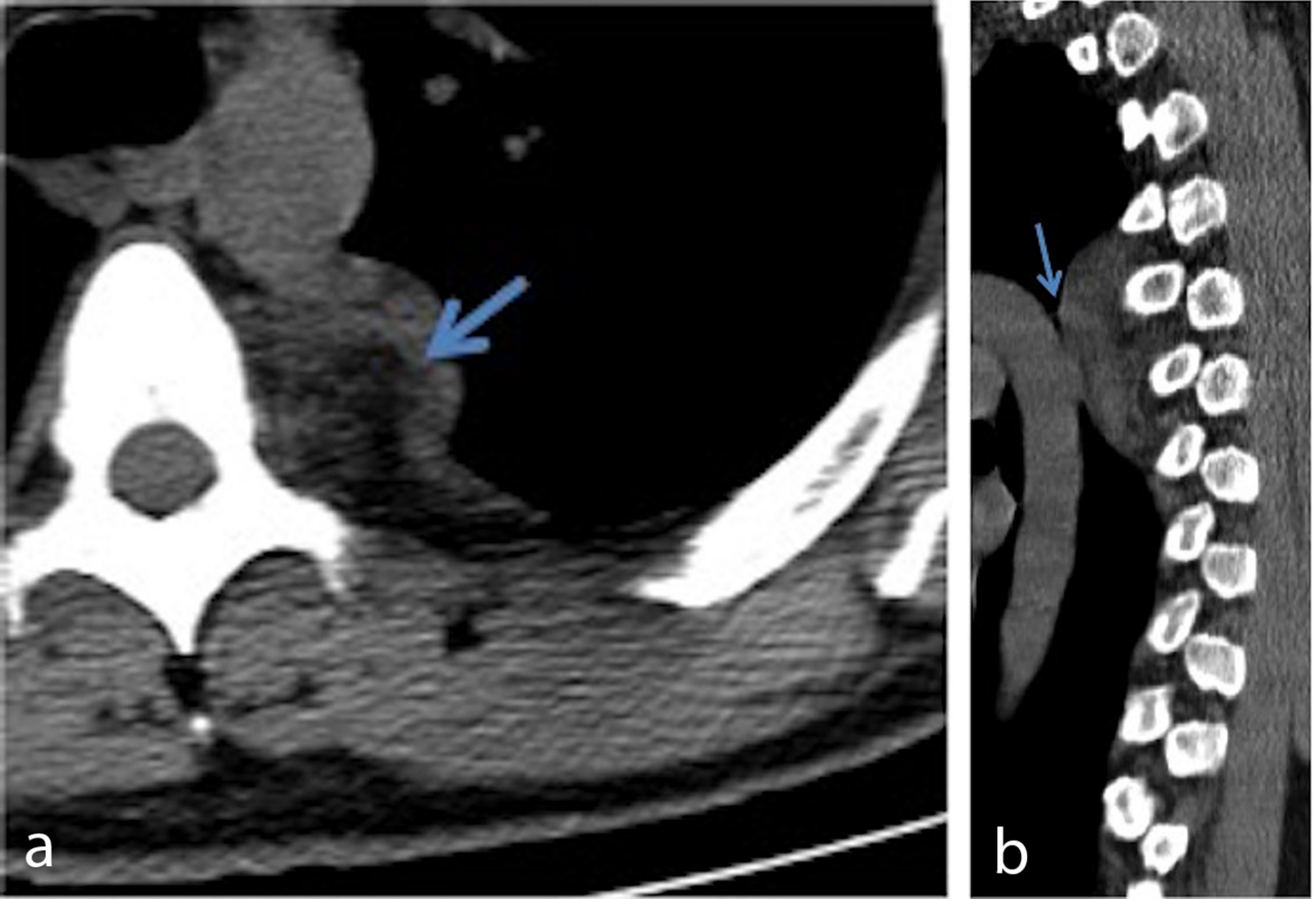
Axial (a) and sagittal (b) non-contrast CT scan shows a well-demarcated left paraspinal mass, abutting the posterior aspect of the descending thoracic aorta (arrow in b) The central portion of the mass has a fatty density (arrow in a).

**Figure 3.**
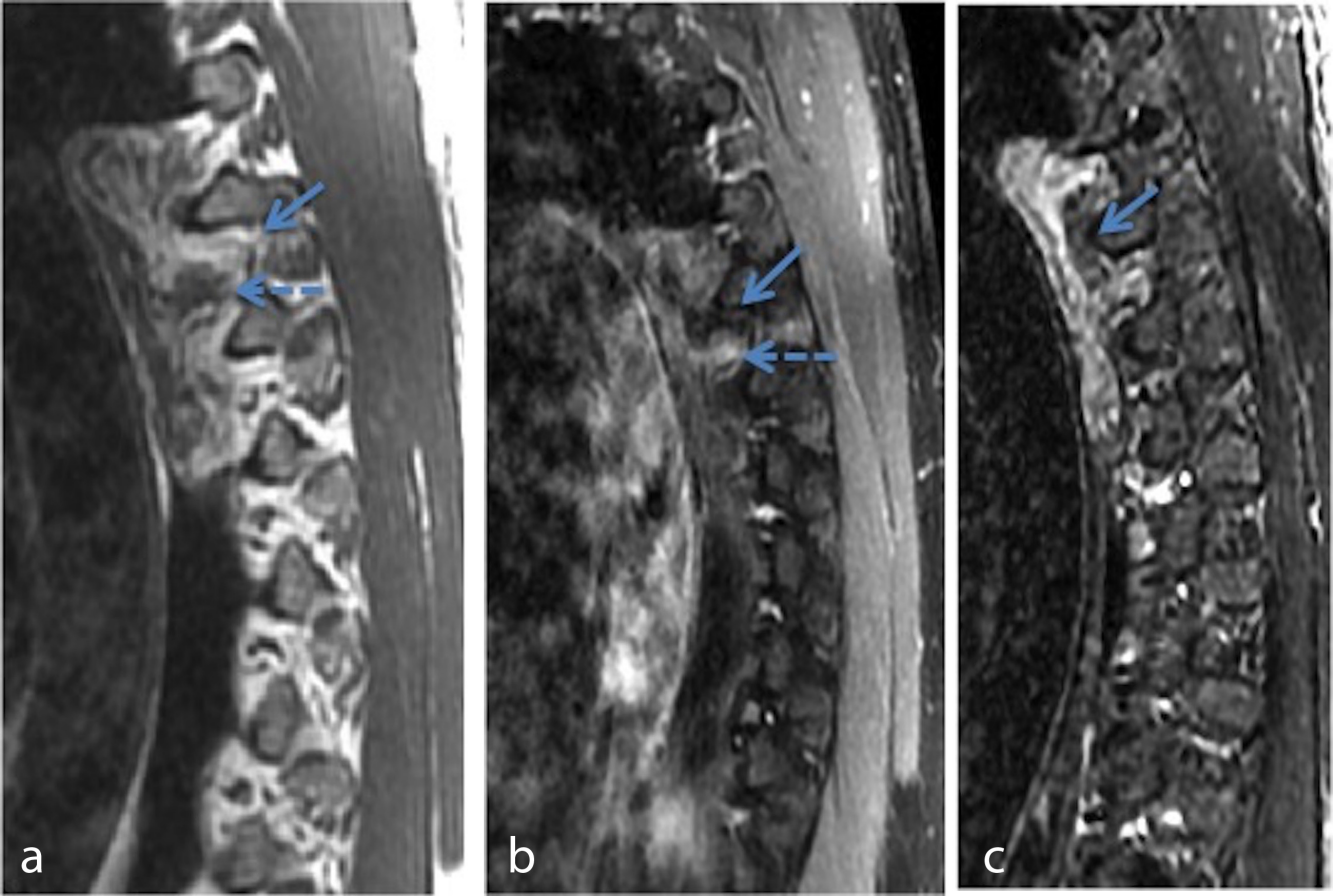
Sagittal *T*_1_ (a) sagittal *T*_1_ postcontrast with fat suppression (b) and sagittal STIR images show a left paraspinal mass with a fatty component. The inherent *T*_1_ hyperintensity of the fatty component of this lesion (solid arrow in a) suppresses on both the postcontrast fat sat (solid arrow in b) and the STIR images (solid arrow in c). The soft tissue component (dashed arrow in a) of the mass shows heterogeneous enhancement (dashed arrow in b). STIR, short tau inversion-recovery.

**Figure 4.**
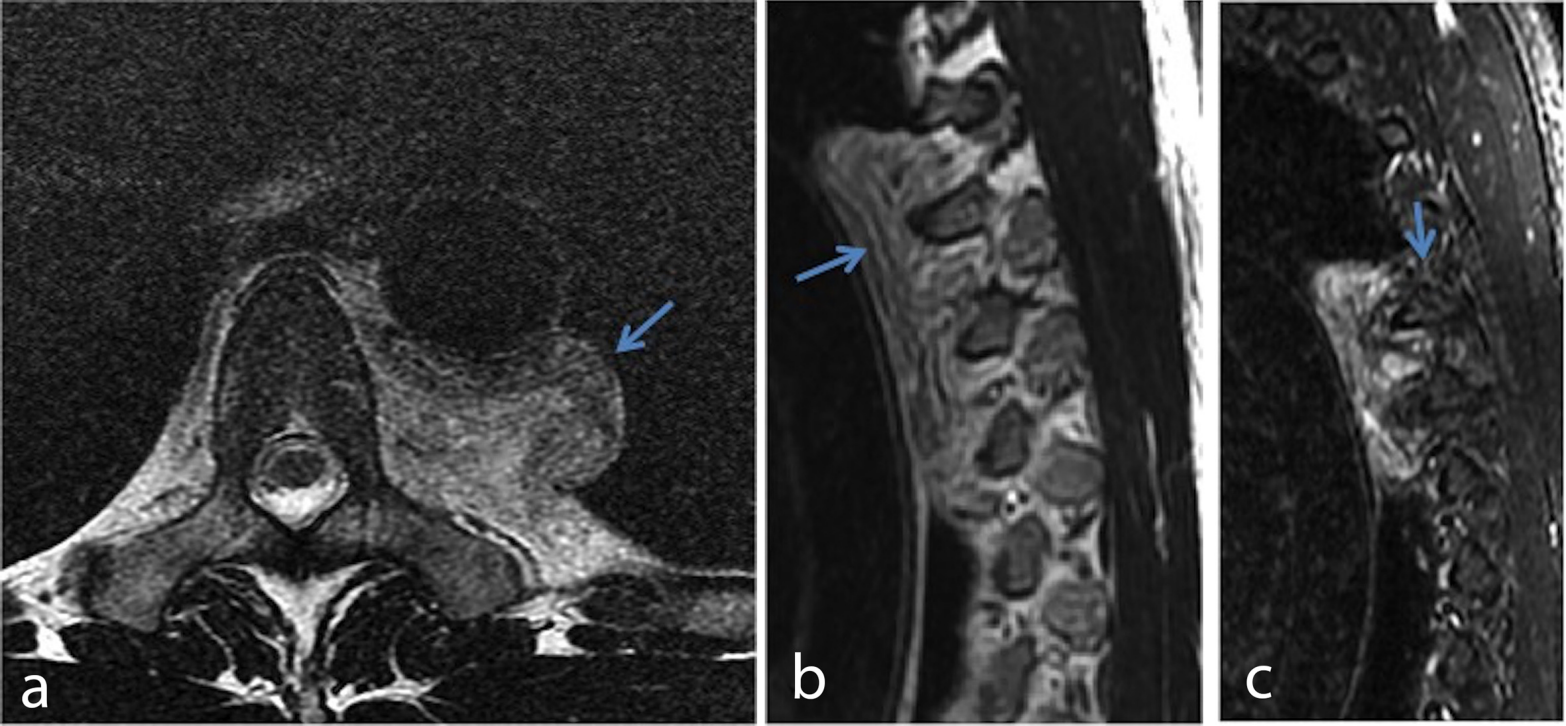
(a) Axial *T*_2_ weighted image shows the well-demarcated margin of the left paraspinal mass (arrow) abutting the descending thoracic aorta. (b) Sagittal *T*_2_ weighted image shows the whorled appearance of the mass. (c) Sagittal short tau inversion-recovery image shows that the mass causes no reactive changes or invasion of the adjacent ribs (arrow).

The scout film obtained during the CT scan of the chest showed a lesion that obscured the normally seen left supralateral contour of the aortic arch (Figure 1). An unenhanced CT scan of the chest demonstrated a well-circumscribed left paraspinal mass measuring 3.3 × 5.6 × 9.2 cm in the transverse, anterioposterior and craniocaudal diemnsions respectively, abutting the descending thoracic aorta and the posterior left fifth through seventh ribs (Figure 2). The mass had heterogeneous attenuation. The relative density of the central portion of the mass was consistent with that of fat. A few punctate foci of calcifications were present within the peripheral soft tissue component. A pre- and postcontrast MRI of the thoracic spine showed a mass abutting the posterior surface of the descending thoracic aorta in the left paravertebral groove, extending from *T*_4_ to *T*_7_ without expansion of or extension into the neural foramina. The inherent *T*_1_ shortening of the central portion of the lesion was suppressed with fat suppression techniques, confirming the central fatty component (Figure 3). The peripheral portion demonstrated mainly intermediate-to-low signal intensity on *T*_1_ weighted images and intermediate signal intensity on *T*_2_ weighted images. The peripheral soft tissue components of the mass showed heterogeneous enhancement after the administration of intravenous gadolinium-based contrast. There was no evidence of bony erosion, reactive oedema or remodelling in either CT or MRI scan (Figure 4).

## Differential diagnosis

The well-defined margins and absence of aggressive features narrowed the differential diagnosis to entities such as ganglioneuroma, schwannoma, angiolipoma and low-grade liposarcoma.

## Treatment

Given the large size of the lesion and the presence of associated chest wall pain, a robotic-assisted thoracoscopy and excision of the mass was performed. After the pleura was incised, a predominantly fatty consistency lesion became apparent. The mass was dissected off the chest wall and completely resected. No difficulties were reported by the surgeon in resecting the mass.

## Outcome and follow-up

**Figure 5.**
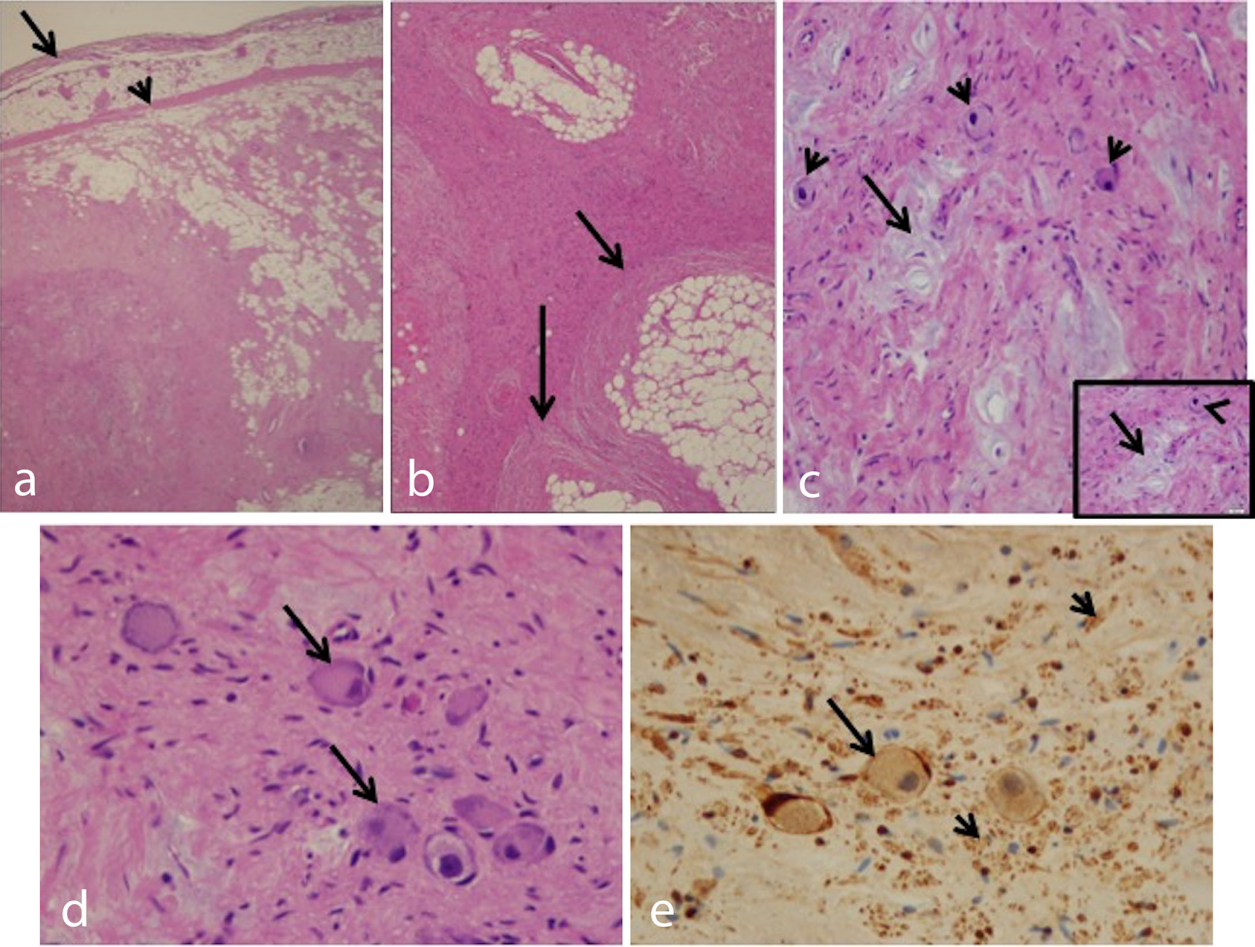
(a) Low power view of a solid nodule under the pleura (black arrow), surrounded by mature adipose tissue and a pseudo capsule (arrow head) (H&E , magnification ×20). (b) Neural bundles (arrows) encasing mature adipose tissue (H&E, magnification ×20). (c) Perivascular mucoid hypocellular bluish material (arrows) was seen within the solid areas adjacent to large mature looking neurons with abundant eosinophilic cytoplasm and eccentrically located nucleus consistent with ganglion cells (arrowheads) (H&E, magnification ×200; insert ×400). (d) Clusters of ganglion cells (arrows) in neural background (H&E, magnification ×400). (e) S100 immunohistochemical stain highlights the presence of ganglion cells (arrow) and neural tissue in the background (arrow heads) (H&E, magnification ×400). H&E, hematoxylin and eosine.

Histological sections of the 36 g mass (2.5 × 7.1 × 7.4 cm) revealed a pseudoencapsulated subpleural heterogeneous lesion. Based on our experience as well as prior reports, the degree of the overestimation of the size of the mass by MRI scan as compared with the measurement of the resected mass stated in the pathology report is customary.^1^ The lesion was composed of neural and fibrous nodules with focal mucoid areas and clusters of ganglion cells intermixed with mature adipose tissue (Figure 5). The neural tissue occasionally surrounded the mature fat in a nodular fashion (Figure 5b). Numerous clusters of ganglion cells were seen in the background of nerve fibres as highlighted by immunohistochemical stain S100 (Figure 5e).

The post-operative course was uneventful and the patient left the hospital on the next day after the procedure. At a six month follow-up, the patient was doing well, except for hyperesthesia at the operative site.

## Discussion

Ganglioneuromas are slow growing tumours of autonomic ganglia. They are typically asymptomatic and often an incidental finding. Clinical manifestations are usually secondary to the location of the neoplasm. Ganglioneuromas most commonly occur in the posterior mediastinum (60–80%); other sites include the retroperitoneum and less commonly the adrenal medulla.^2,3^ Ganglioneuromas are responsible for up to 35% of the intrathoracic neurogenic tumours.^4,5^

Posterior mediastinal ganglioneuromas with fatty components as described here are rare. A review of the English literature revealed only five reported cases (Table 1).^6–10^ In addition to these case reports, two different retrospective studies described four^11^ and two^12^ cases of mediastinal and thoracic ganglioneuroma, respectively, containing variable amounts of fatty tissue. Two potential aetiologies have been proposed to explain the presence of fatty component in this type of ganglioneuromas. One theory suggests that the fatty component arises from involvement of paravertebral fat.^11^ Alternatively, ganglioneuroma may undergo fatty degeneration. The latter explanation can also account for the older mean age of patients presenting with fat containing ganglioneuromas. The average age of these patients is typically reported to be in the mid-forties, which is higher than the typical age for ganglioneuromas.^4,6,8,10,12^

**Table 1.** The case reports in the English literature reporting the ganglioneuroma with the fatty component.

References	Age (years)	Sex	Clinical presentation	Location and orientation	Size	Special imaging findings	Enhancement
Hara et al^6^	54	Female	Incidental	Left paravertebral (craniocaudal)	11 × 3 × 6.5 cm	Whorled appearance on CT scan	Minimal
Demir et al^7^	33	Male	Scoliosis	Right paravertebral (*T*_6_–*T*_11_) (cranicaudal in the images)	–	Scattered fatty areas, calcifications and vertebral scalloping	Intense
Yorita et al^8^	66	Female	Incidental	Left paravertebral (*T*_7_–*T*_9_) craniocaudal	l2 × 6 × 4 cm	Rich in fat, especially in peripheral areas	Slight-to-mild heterogeneous
Duffy et al^9^	27	Female	Incidental	Right paravertebral (*T*_9_–*T*_12_)(craniocaudal)	Incidental	The mass was effacing the right side of the cord and displacing it slightly towards the left	Some enhancement in the areas of intermediate SI
Ko et al^10^	53	Female	Incidental 9 × 4.5 × 10.0	Right paravertebral (GT4–T4)	Incidental	The tumour crossed into the left posterior mediastinum	The soft tissue component enhanced minimally

Limited reported cases of lipomatous ganglioneuromas make generalisations about the imaging findings of these rarely reported tumours difficult. Furthermore, excluding low-grade liposarcoma from the differential diagnosis of mediastinal fatty mass without aggressive features can also be challenging. Independent of the fatty components, several cases of the gangalioneuroma reported whorled appearance on both *T*_1_ and *T*_2_ weighted images, a feature which we also observed.^13^ Punctate calcifications, as seen in our case, have also been reported with these types of tumours.^14^ The oblong shape of this mass with craniocaudal orientation (Figure 2) is another potential clue to the diagnosis and the benign nature of these tumours. This craniocaudal orientation was observed in three of the other reported cases.^6,8,9^

Radiological–pathological comparisons by Forsythe et al^2^ demonstrates that the degree and heterogeneity of enhancement corresponds to the proportion of components such as myxoid stroma, cellular components and collagen fibres.

The case we describe here, along with the other case reports, justify the inclusion of ganglioneuromas in the differential diagnosis of posterior mediastinal masses with fatty component. Features such as the craniocauadal orientation, punctate calcification and whorled appearance should further narrow the differential consideration to this entity. Continued study and reporting of additional lipomatous ganglioneuromas may help further characterize lipomatous ganglioneuromas and guide treatment plans.

## Treatment and prognosis

The prognosis for ganglioneuromas is favourable. Surgical removal is the treatment of the choice, as the diagnosis of ganglioneuroma cannot be ascertained before the removal of the mass. Although rare, spontaneous development of malignant peripheral sheath tumours in a benign ganglioneuroma has been reported.^15,16^

## Learning points

1. Ganlioneuromas should be included in the differential diagnosis of fat containing posterior mediastinal masses.
2. Craniocaudal orientation, intrinsic whorled appearance and punctate calcification should favour the diagnosis of ganglioneuroma.
3. The ganglioneuromas with fat typically present in middle-aged adults, a mean age that is older than the typical age of presentation for more common forms of ganglioneuroma.

## Consent

Written informed consent for the case to be published (incl. images, case history and data) was obtained from the patient for publication of this case report, including accompanying images.
